# Exploring the correlation between Drp1 protein and the neurotoxicity of propofol

**DOI:** 10.3389/fnins.2025.1614362

**Published:** 2025-07-21

**Authors:** Pu Guo, Jing Mei

**Affiliations:** ^1^Graduate School of Xinjiang Medical University, Urumqi, Xinjiang, China; ^2^Department of Anesthesiology, People’s Hospital of Xinjiang Uygur Autonomous Region, Urumqi, Xinjiang, China

**Keywords:** Drp1, propofol, neurotoxicity, mitochondria, neuroprotection

## Abstract

Neurotoxicity is a common toxic reaction associated with the use of the anesthetic drug propofol. With the widespread use of propofol, the issue of neurotoxicity has garnered significant attention. Mitochondria are the energy metabolism centers of cells and play a crucial role in biological processes such as cell growth and development, invasion and metastasis, division and differentiation, and apoptosis. Dynamin-related protein 1 (Drp1) is a key regulator of mitochondrial fission that can modulate the dynamic balance of mitochondria and plays an important role in maintaining mitochondrial morphology and function. The abnormal expression of Drp1 is closely related to the occurrence and development of various pathological conditions. Through a systematic review of multi-species animal and cellular studies, we elucidated the correlation between Drp1 and propofol-induced neurotoxicity. By analyzing Drp1-mediated mitochondrial fragmentation across different organ systems, this work provides crucial theoretical foundations for developing Drp1-targeted strategies in propofol neurotoxicity detection, prevention, and pharmacological intervention.

## Graphical abstract

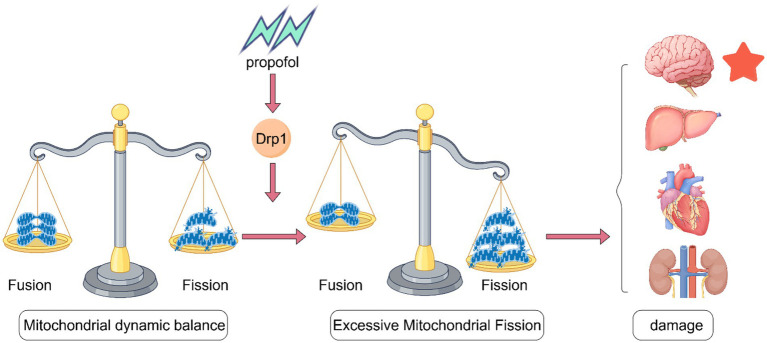

## 1 Introduction

Neurotoxicity refers to damage to the nervous system, which can lead to various clinical manifestations such as altered consciousness, motor function impairment, and cognitive decline. Neurotoxicity can be caused by a variety of factors, including drugs, toxins, and infections (Della Giovampaola et al., 2022). The neurotoxic mechanism of propofol mainly induces apoptosis, but the specific mechanism of its action is currently unclear and requires further research. The protein Drp1 plays a central role in regulating mitochondrial morphological changes, especially when cells respond to various physiological and pathological stimuli. Studies have shown that excessive activation of the Drp1 can lead to excessive mitochondrial fission, resulting in dysfunctional mitochondria, which in turn triggers apoptosis and tissue damage (Wang and Qiu, 2023; Zhang B.-F., et al., 2024). The dynamic balance of mitochondria relies on the coordination of fission and fusion processes. Drp1, by promoting mitochondrial fission, can help cells adapt to changes in energy demand and respond to challenges such as oxidative stress (Rosdah et al., 2020; Yang et al., 2022). The balance between mitochondrial fusion and fission is disrupted in hippocampal neuron injuries, and the main protein involved, Drp1, is linked to neurodevelopment, neural plasticity, and neurological issues (Gao et al., 2022). Drp1’s role in mitochondrial fission is essential for repairing injuries in hippocampal neurons. This article reviews the structure and function of Drp1, discusses its role in propofol neurotoxicity based on animal studies, examines the mechanistic link between Drp1 and neurotoxicity, analyzes Drp1’s involvement in mitochondrial dysfunction across different organs, and suggests targeting Drp1 as a strategy to prevent neurotoxicity. In summary, the discussion in this article provides important insights for the detection, prevention, and drug development of propofol-induced neurotoxicity from the perspective of Drp1.

## 2 Structure and function of Drp1 protein

Drp1 is an important GTPase that primarily participates in the division and dynamic regulation of mitochondria. Drp1 has multiple protein isoforms, which arise from the alternative splicing of pre-mRNA transcripts encoded by a single gene. The structure of Drp1 mainly consists of a GTPase domain, a GTP-binding domain, and a C-terminal helical domain. At its N-terminus, Drp1 also has a proline-rich region. The GTPase activity of Drp1 enables it to bind GTP and drive the mitochondrial division process through GTP hydrolysis. The domains of Drp1 play a crucial role not only in its self-assembly and activity regulation but also in regulating mitochondrial morphology and function through interactions with other proteins. Drp1 interacts with mitochondrial fission proteins such as Fis1, Mff, MiD49, and MiD51 (Nolden et al., 2023). For example, Drp1 promotes the process of mitochondrial fission by binding to adaptor proteins (such as Mff and Fis1) on the outer mitochondrial membrane through its C-terminal helical domain (Nolden et al., 2023; Torcasio et al., 2024). Research shows that the direct interaction between Fis1 and Drp1 can maintain mitochondrial morphology, with Fis1 influencing the balance of mitochondrial fission and fusion by regulating the assembly state of Drp1 (Choi et al., 2020; Egner et al., 2022). Drp1 is involved in the process of mitochondrial fission, including recruitment from the cytosol to the outer mitochondrial membrane (OMM), high-level self-assembly, GTP hydrolysis, and eventual fragmentation (Feng et al., 2020).

In recent years, it has been reported that the function of Drp1 is not limited to mitochondrial fission but also involves various biological processes in cells, including cellular stress responses, metabolic regulation, and apoptosis. There is evidence that mitochondria in the spinal dorsal horn (SDH) are sensitive to neuropathic pain (NP), and targeting mitochondrial Drp1 overexpression alleviates pain hypersensitivity, providing a new therapeutic target for pain treatment (Zhang K.-L., et al., 2022).

Abnormal levels or dysfunction of Drp1 are closely linked to the development of several diseases, including neurodegenerative disorders, cardiovascular diseases, and cancers (Zeng et al., 2020). Because the nervous system requires a large amount of energy, mitochondrial damage or dysfunction greatly affects neuronal activity and can even lead to their death.

## 3 Propofol and neurotoxicity

### 3.1 The mechanism of apoptosis-induced neurotoxicity

**Figure 1:**
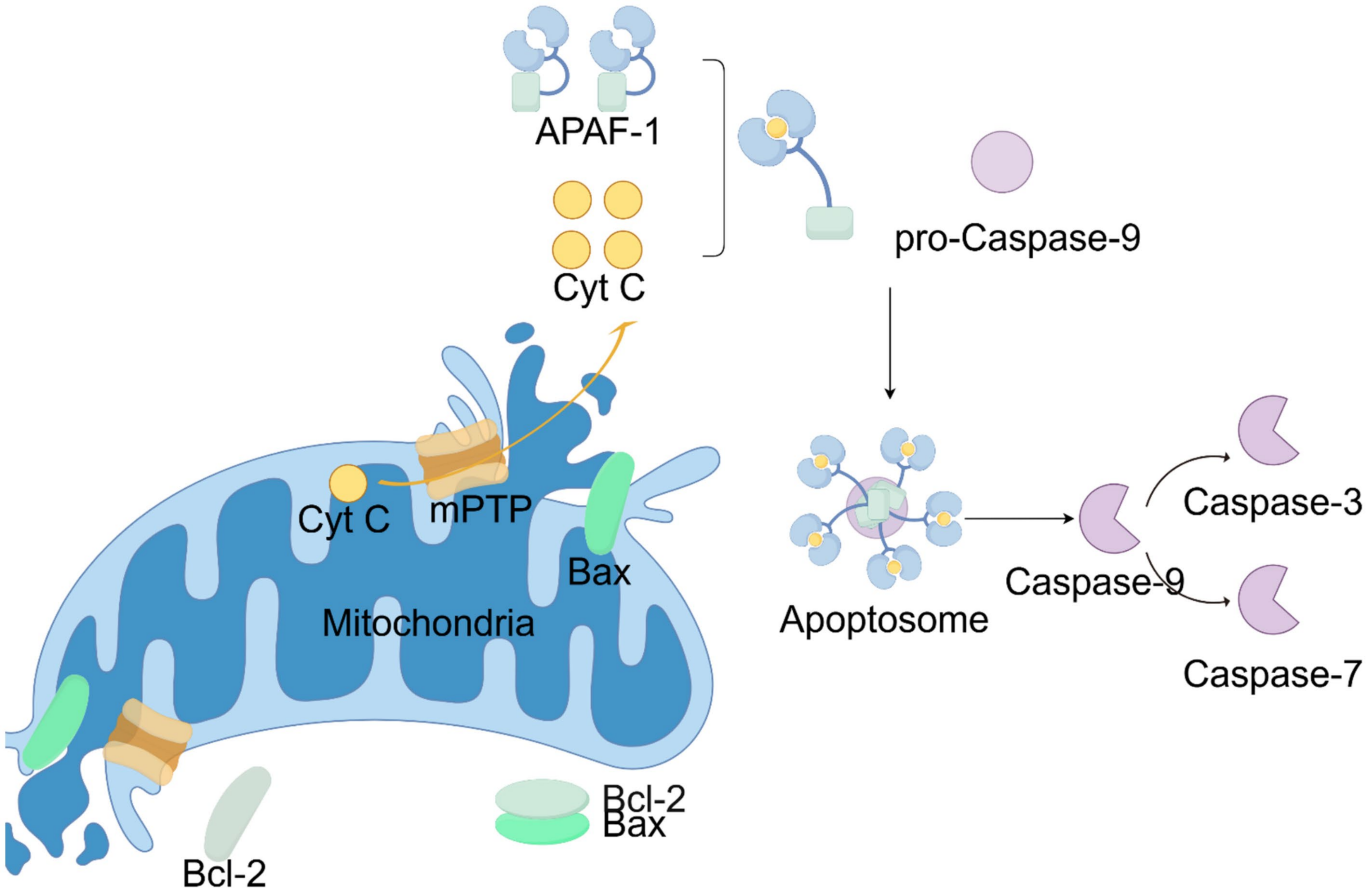
The basic process of cell apoptosis. Created with Figdraw.

Propofol enhances the effects of the inhibitory neurotransmitter gamma-aminobutyric acid (GABA) by binding to the β+/α-subunits of the GABAA receptor, leading to hyperpolarization of the neuronal membrane and reduced neuronal excitability. This allows propofol to rapidly induce loss of consciousness and maintain anesthesia. Propofol may also regulate the balance of excitation and inhibition in the brain by affecting other neurotransmitter systems, such as the glutamatergic system (Li et al., 2020). The apoptotic pathway induced by propofol is an important aspect of studying its neurotoxic mechanisms. Propofol can lead to the apoptosis of nerve cells and negatively affect their survival. Apoptosis is a programmed pattern of cell death that typically occurs under stress conditions. At this time, the permeability of the mitochondrial membrane increases, and the membrane potential decreases, leading to the release of cytochrome C into the cytoplasm (Gottlieb et al., 2003), which in turn activates downstream caspases. This process is usually triggered by the opening of the mitochondrial permeability transition pore (mPTP). The opening of mPTP is associated with various factors, including calcium ion concentration, oxidative stress, and changes in the mitochondrial internal environment (Li et al., 2024). The respiratory chain consists of four multi-protein complexes (I, II, III, IV), two mobile electron carriers, ubiquinone (coenzyme Q), and cytochrome C. Propofol inhibits mitochondrial complexes II and III in the hippocampus. This inhibition can impair electron transfer and reduce the mitochondrial membrane potential (Finsterer and Frank, 2016). More and more studies have shown that propofol promotes changes in the expression of apoptosis-related proteins such as Bcl-2 and Bax, thereby affecting cell survival (Xu et al., 2022). For instance, Maternal propofol exposure during mid-gestation significantly increased Cleaved-Caspase3 levels and pro-apoptotic Bax expression while decreasing anti-apoptotic Bcl-2 in fetal rats, ultimately resulting in cognitive deficits (particularly in learning and memory functions) in male offspring (Mei et al., 2022a). A study found that low concentrations of propofol can promote the survival of nerve cells, while high concentrations of propofol significantly increase the rate of cell apoptosis, demonstrating a concentration-dependent effect (Xu et al., 2022) (Figure 1).

#### 3.1.1 MAPK and cell apoptosis

Propofol promotes neuronal apoptosis by inhibiting the MAPK/ERK signaling pathway, disrupting the dynamic balance between pro-apoptotic and anti-apoptotic signals. Studies have found that in human neuronal SH-SY5Y neuroblastoma cells and rat hippocampal tissue cells, the levels of ERK1/2 and its phosphorylated forms (pERK1/2) significantly decrease after propofol treatment, while the phosphorylation level of NF-κB p65 also declines. Propofol treatment also leads to the upregulation of microRNA-34a, which can directly target and inhibit the expression of key molecules in the MAPK/ERK pathway (such as ERK1/2), forming a positive feedback mechanism that further exacerbates ERK signal inhibition (Li et al., 2018).

Phosphorylated CREB is an important survival signaling molecule downstream of the ERK signaling pathway and plays a role in the apoptosis of nerve cells induced by propofol. In early animal experiments, we observed that propofol anesthesia applied to rats during mid-pregnancy resulted in cognitive impairments in their offspring. A key mechanism behind this phenomenon is the activation of histone deacetylase 2 (HDAC2), which in turn inhibits the CREB and NMDA receptor subtype (NR2B) signaling pathway, leading to impaired synaptic plasticity in the hippocampus. Over-phosphorylation of HDAC2 can reduce the phosphorylation level of CREB, thereby decreasing the expression of CREB protein (Mei et al., 2022c).

p38MAPK, as a member of the MAPK family, can be activated by stress factors such as pro-inflammatory cytokines and toxins, leading to inhibition of cell growth and increased apoptosis. The action of propofol can activate the p38MAPK signaling pathway, thereby reducing the vitality of nerve cells and increasing the occurrence of apoptosis (Xu et al., 2020).

#### 3.1.2 Mitochondria and cell apoptosis

Propofol affects a series of mitochondrial-related proteins, including Cyclin-dependent kinase 1 (CDK1), phosphorylated dynamin-related protein 1 (p-DRP1), Parkin1, and DJ-1, by inhibiting Pink1 in developing neurons. This inhibitory effect leads to an increase in the expression levels of these proteins under propofol exposure, resulting in mitochondrial deformation, vacuolization, and swelling, ultimately causing dysfunction, which affects the proliferation, differentiation, and apoptosis of nerve cells, leading to neurotoxicity (Liang et al., 2021). In the CNS, glucose enters neurons through glucose transporter 3 (GLUT3), and then within the cell, mitochondria convert glucose into ATP through oxidative phosphorylation. However, exposure to propofol leads to a limitation in the energy supply of hippocampal neurons, primarily mediated by the downregulation of GLUT3 protein expression, which affects glucose uptake and transport (Xiao et al., 2020). This not only increases the levels of oxidative stress but also promotes the generation of reactive oxygen species (ROS), further exacerbating the damage to nerve cells (Zhang W. et al., 2022).

#### 3.1.3 Ferroptosis and apoptosis

Ferroptosis is a programmed cell death mechanism distinct from apoptosis. Research indicates that propofol may cause an imbalance in the production and degradation of lipid reactive oxygen species within cells. This imbalance can activate ferroptosis, leading to neuronal apoptosis and resulting in neurotoxicity (Chen J. et al., 2023). In the early stages of iron overload, ferroptosis is triggered through the p53 pathway, while in the later stages, it leads to apoptosis accompanied by mitochondrial dysfunction (Zhang, 2020). Thus, ferroptosis and apoptosis may occur sequentially or synergistic regulatory relationship.

#### 3.1.4 Long non-coding RNA and cell apoptosis

Long non-coding RNAs (lncRNAs) play pivotal roles in transcriptional regulation and chromatin remodeling complex formation, and are also capable of modulating mRNA and protein expression levels. Exposure to propofol leads to changes in lncRNA, mRNA, and their downstream synergistic signaling networks. These lncRNAs co-express with calcium signaling pathways (such as CaMKII) and genes related to synaptogenesis, which may trigger neurotoxic responses in mice (Logan et al., 2018). However, the specific mechanisms remain to be further studied. In summary, apoptotic mechanisms play an indispensable role in the pathogenesis of neurotoxicity. The pathological process of apoptosis is intricate, but understanding it offers valuable insights into investigating the neuroprotective effects.

### 3.2 Neurotoxicity of propofol in different developmental stages of the population

The timing of synaptogenesis in humans ranges from late pregnancy to the third year after birth. During this developmental stage, propofol can easily cross the blood–brain barrier, leading to neurotoxic effects on the developing brain. This toxicity primarily manifests as damage to nerve cells, which may negatively impact learning and memory abilities (Zhang W., et al., 2024; Twaroski et al., 2015).

The mid-pregnancy period is a critical time for neurogenesis and neuronal migration, during which the fetus’s sensitivity to external environmental factors significantly increases. Based on previous animal experiments conducted by our research group, we found that maternal rats exposed to propofol during mid-gestation gave birth to offspring showing significantly prolonged escape latency and reduced platform crossings in behavioral tests. Additionally, the number of neurons in the hippocampal region was significantly decreased, while the expression levels of Bax, Cleaved-Caspase3, and CaMKII were increased, and the expression of Bcl-2 in the hippocampus was suppressed (Mei et al., 2022a).

In the pediatric stage, there is a period known as the “danger window,” which refers to a specific age group where exposure to multiple general anesthetics (mGA) may lead to long-term neurocognitive disorders. It is generally believed that the most sensitive period is before the age of 3 or 4. During this developmental stage, the brain undergoes significant remodeling processes, and anesthetic agents may interfere with the mechanisms of synaptogenesis, neurogenesis, and cell survival in neurons (Colletti et al., 2023), thereby affecting long-term learning and memory capabilities.

For patients aged 60 and above, the incidence of postoperative cognitive dysfunction is more than twice that of patients under 60. This may be related to the fact that elderly patients typically face more risk factors for neurovascular diseases, increased white matter damage, and reduced cognitive reserve (Berger et al., 2015).

In patients with pre-existing neurological disorders, such as Parkinson’s disease and Alzheimer’s disease, the use of propofol may further exacerbate their neurological impairment or accelerate disease progression. In Alzheimer’s patients, the interaction between intrabrain Aβ plaques and hyperphosphorylated Tau protein synergizes with the effects of propofol: propofol promotes Tau phosphorylation by activating GSK3β, while abnormal Tau further inhibits mitochondrial transport, creating a vicious cycle (Eryilmaz et al., 2024). In patients in the preclinical stage of Parkinson’s disease, propofol may worsen the damage to mitochondria caused by α-synuclein oligomers. Overall, the neurotoxicity of propofol varies across different developmental stages. Understanding these differences.

### 3.3 Neurotoxicity of propofol in different types of nerve cells

The effects of propofol on inhibitory synaptic currents vary across different brain nuclei, indicating that it may have specific effects in different types of neurons (Liu et al., 2021). Astrocytes are the most common glial cells in the central nervous system and play important roles in overall brain metabolism, synaptic transmission, and neuronal protection (Gollihue and Norris, 2020). Astrocytes are responsible for providing energy and metabolic support to neurons by releasing extracellular vesicles. Studies have found that aging, injury, and disease can lead to mitochondrial dysfunction in astrocytes, which can have harmful effects on neurons. Co-culturing neurons with propofol and astrocytes with propofol revealed that propofol reduces neuronal viability and ATP levels, increases neuronal death rates, decreases mitochondrial membrane potential, and increases ROS levels and apoptosis rates, indicating that propofol-induced mitochondrial damage in neurons is more pronounced compared to astrocytes (Zhou et al., 2024), and that the two types of cells respond differently to the neurotoxicity of propofol.

## 4 The systemic toxicity of propofol and the role of Drp1 in mitochondrial dysfunction in different organs

Propofol’s toxic reactions extend beyond the nervous system, primarily causing dose-dependent suppression of the cardiovascular and respiratory systems, with symptoms such as hypotension, bradycardia, and apnea. An overdose can result in significant hypotension, arrhythmias, or even cardiac arrest. Moreover, prolonged high-dose use of propofol (exceeding 4 mg/kg/h) for over 48 h may trigger propofol infusion syndrome (PRIS), characterized by severe conditions such as metabolic acidosis, rhabdomyolysis, hyperkalemia, and multiple organ failure (Singh and Anjankar, 2022). Other possible adverse reactions include injection pain, allergic reactions, and, although rare, lipid metabolism disorders that can be fatal. The risk of toxicity significantly increases in children, critically ill patients, and with prolonged use, necessitating strict monitoring of vital signs and medication dosage. The corresponding toxic reactions of propofol on different organs are as follows.

Propofol is mainly metabolized in the liver, relying on hepatic blood flow, though extrahepatic metabolism also occurs (Kazi et al., 2025). In adults, UGT1A9 primarily mediates hepatic metabolism, whereas in neonates, cytochrome P450 plays a key role (Sandra et al., 2021). The use of propofol can produce ROS that overwhelm the body’s antioxidant defenses, leading to oxidative stress and potential liver damage. Research indicates that propofol doses exceeding clinical levels (e.g., an IC₅₀ of 254.904 μg/mL) can be toxic to hepatic fibroblasts (AML12) due to elevated intracellular ROS, which triggers mitochondrial pathways that lead to apoptosis (Pehlivan et al., 2023). Mitochondrial fission plays a crucial role in maintaining hepatocyte homeostasis and preventing liver tumorigenesis (Ma et al., 2025).

When activated, Drp1 moves to the mitochondria in cardiac cells, leading to significant mitochondrial division. The excessive fragmentation of mitochondria disrupts the structure of the heart and affects blood flow. This disruption can lead to multi-organ dysfunction and higher mortality rates (Liu et al., 2024). The main pathophysiological mechanism behind PRIS is the disruption of the mitochondrial respiratory chain, which hampers ATP synthesis and leads to cellular hypoxia. For example, propofol causes coenzyme Q-sensitive leakage in the mitochondria of cardiac myocytes. This leakage uncouples immature cardiomyocytes and disrupts coenzyme Q’s role in the electron transport chain (Barajas et al., 2022).

Furthermore, human embryonic kidney cells (HEK-293) serve as an excellent *in vitro* model for examining renal cell physiology. Studies indicate that with an increase in propofol dosage, there is a corresponding decline in cell viability alongside an increase in ROS, culminating in cell apoptosis and ultimately cell death (Pehlivan et al., 2024). Similar trends have been observed in other cell types, such as human promyelocytic leukemia cells, where elevated doses of isoflurane also hinder oxygen utilization and initiate apoptosis. The extent to which propofol exhibits either complete or primarily mitochondrial toxicity remains uncertain, particularly in individuals with mitochondrial defects. However, it can be inferred that in patients with pre-existing mitochondrial disorders, propofol may further aggravate mitochondrial dysfunction (Finsterer and Frank, 2016).

In summary, propofol causes varying degrees of damage to the nervous system, liver, heart, and kidneys. A deeper exploration of the effects of propofol on different systems and organs provides important clues for our comprehensive understanding of the toxic reactions of propofol.

## 5 The mechanism by which Drp1 affects the neurotoxicity of propofol

The mitochondrial quality control system, which includes mitochondrial fusion and fission, mitophagy, and mitochondrial biogenesis, is crucial for maintaining mitochondrial homeostasis and function (Eldeeb et al., 2022). Mitochondrial fission is regulated by a series of proteins, with Drp1 being the dominant one. Overexpression of Drp1 leads to excessive mitochondrial fission, resulting in dysfunctional mitochondria. In neurons, abnormal mitochondrial fission is closely related to the occurrence of various neurodegenerative diseases such as Alzheimer’s disease (Joshi et al., 2018), Huntington’s disease (Roe and Qi, 2018), and Parkinson’s disease (Zhang et al., 2020). Therefore, inhibiting the overactivation of Drp1 or regulating mitochondrial dynamics is a method to promote neuroprotection (He et al., 2024).

### 5.1 Phosphorylation state of Drp1 and neurotoxicity

The activity and function of Drp1 are regulated by various post-translational modifications (PTMs), including phosphorylation, SUMOylation, ubiquitination, palmitoylation, S-nitrosylation, and O-GlcNAcylation (He et al., 2024). Among numerous studies, the phosphorylation modification of Drp1 has attracted particular attention. Specifically, serine 616 (S616) and serine 637 (S637) in Drp1 have been identified as the main phosphorylation sites. S616 can promote mitochondrial fission (Cho et al., 2013), while S637 promotes mitochondrial fusion. Cyclins and cyclin-dependent kinases (CDKs) regulate the phosphorylation of Drp1 Ser616, which then activates Drp1, leading to oligomerization at the fission sites of the mitochondrial outer membrane and initiating mitochondrial constriction and division, facilitating the transition of the cell from G1 to S phase (Dasgupta et al., 2020). A recent study showed that after co-culturing with propofol, propofol treatment significantly increased the phosphorylation level of Drp1 at the Ser616 site in neurons, resulting in enhanced mitochondrial fission and the formation of a fragmented mitochondrial network, ultimately triggering neurotoxicity (Zhou et al., 2024). PINK1 can mediate Drp1 S616 phosphorylation to promote mitochondrial fission, thereby regulating the development and maturation of excitatory circuits and synaptic plasticity in the mouse hippocampus and cortex (Gao et al., 2022). Additionally, PINK1 also influences mitophagy.

There is a significant association between the post-translational modifications of Drp1 and neurodegenerative diseases. For example, blocking Drp1 Ser579 phosphorylation inhibits Aβ1-42 induced neurotoxicity (Xu et al., 2021); in the brains of AD patients, S-nitrosylation of Drp1 promotes the formation of dimers and enhances GTPase activity. This change accelerates mitochondrial fission, ultimately leading to damage to neuronal synapses or cell death (Cho et al., 2009). Currently, there is a lack of research and relevant information on other post-translational modifications of Drp1, apart from dephosphorylation, in regulating the neurotoxic mechanisms in the propofol exposure model. Therefore, further research should be conducted on the post-translational modifications of Drp1, comparing their similarities and differences in neurodegenerative lesions and the propofol exposure model.

### 5.2 Drp1-mediated signaling pathway and neurotoxicity

In addition, the activity and function of Drp1 are regulated by various intracellular signaling pathways, including AMPK and ERK signaling pathways, which play a key role in cellular metabolic adaptation and survival (Timmins et al., 2024). The translocation of Drp1 to the outer mitochondrial membrane is a necessary step for CaMKII to exert its function. Drp1 can also regulate mitochondrial dynamics through interactions with signaling molecules such as TBK1, thereby affecting the immune response and metabolic state of the cell (Chen et al., 2020). Data indicate that exposure to propofol in human embryonic stem cell (hESC)-derived neurons leads to Drp1 activation via CDK1, increased mitochondrial fission, reduced mitochondrial membrane potential, and opening of the mPTP, all of which collectively induce cell death (Twaroski et al., 2015), but do not significantly alter the expression of mitochondrial fusion-related proteins.

### 5.3 ROS produced by Drp1 and neurotoxicity

By regulating mitochondrial fission, Drp1 can affect intracellular energy metabolism and oxidative stress, leading to apoptosis. For example, overexpression of Drp1 has been found to induce the production of intracellular ROS, activating apoptotic signaling pathways and promoting cell death (You et al., 2020). The activity of Drp1 is also influenced by changes in calcium ion concentration, with calcium overload activating Drp1 (Feng et al., 2020). The activation of Drp1 and the production of reactive oxygen species (ROS) create a vicious cycle: ROS promote the activation of Drp1, while excessive activation of Drp1 leads to the production of more ROS. Increased ROS levels can lead to the rise of inflammatory factors IL-6, TNF-*α*, IL-18, and IL-1β, causing damage to neurons (Chen P. et al., 2023). Propofol inhibits PINK1 levels, increasing ROS production.

### 5.4 The effect of Drp1 and mitophagy on neurotoxicity

Mitophagy refers to the selective recognition and clearance of damaged, dysfunctional, or superfluous mitochondria by cells through the autophagy mechanism. Drp1 interacts with mitochondrial autophagy-related proteins such as PINK1 and Parkin to regulate mitochondrial quality control. In the context of cellular stress and apoptosis, Drp1 promotes mitochondrial fission, increases the occurrence of mitochondrial autophagy, and helps cells eliminate damaged mitochondria, thereby maintaining cell survival (Hasani et al., 2023). Animal studies have found that propofol induces apoptosis in nerve cells but inhibits the mTOR signaling pathway, leading to an increase in autophagy markers LC3-II and Beclin-1, enhancing the level of cellular autophagy and initiating adaptive protective responses in the body (Mei et al., 2022b).

**Figure 2:**
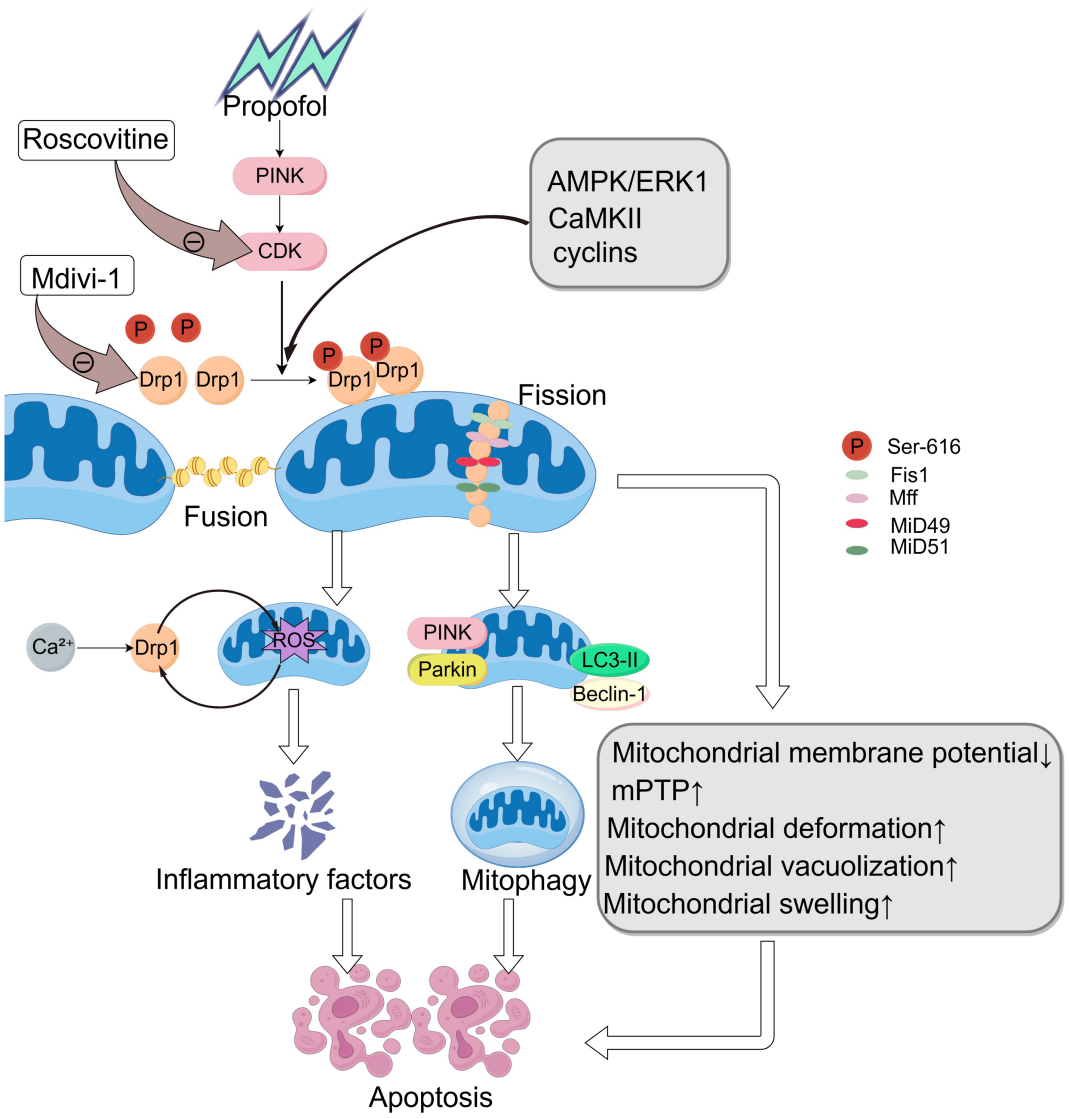
The mechanism of propofol in promoting apoptosis via Drp1 regulation. Created with Figdraw.

In summary, the mechanisms by which Drp1 influences propofol-induced neurotoxicity are not independent; rather, they are interconnected and intricately intertwined (Figure 2).

## 6 Targeting Drp1 as a therapeutic target to prevent propofol neurotoxicity

It is known that astrocytes can release healthy mitochondria to migrate to damaged neurons to alleviate propofol-induced neurotoxicity. Inhibitors of Drp1, such as Mdivi-1, have been shown to weaken the effects of propofol on NSC proliferation, differentiation, apoptosis, and ROS production, as well as mitigate propofol-induced mitochondrial ultrastructural changes and MMP inhibition, effectively reducing pathological changes in neurodegenerative and metabolic diseases (Liang et al., 2021; Furuya et al., 2023; Sun et al., 2024). However, it is noteworthy that pretreatment with Mdivi-1 only partially attenuates, rather than completely reverses, propofol-induced cell death, which may be associated with the potential role of uninvestigated components. In contrast, pretreatment with the CDK1 inhibitor Roscovitine rescues propofol-induced cell death, Drp1 activation, and increased mitochondrial fission (Hogarth et al., 2023). Hypoxic preconditioning can increase the expression of pDrp1 protein and glucose transporters (GLUT1 and GLUT3), providing neuroprotection and promoting brain recovery (Xiao et al., 2020). Pharmacological activation of AMPK using agents such as AICAR effectively suppresses propofol-induced p53 expression and subsequent apoptotic signaling activation, thereby attenuating propofol-mediated neurotoxicity (Xiao et al., 2022). Experimental evidence demonstrates that Liuwei Dihuang Wan enhances cognitive function and confers neuroprotective effects against brain aging by modulating mitochondrial dynamics. Specifically, LDW upregulates mitochondrial fusion proteins while downregulating Drp1 expression, thereby attenuating mitochondrial impairment and restoring the balance between mitochondrial fusion and fission (Wang et al., 2023).

## 7 Future research directions and challenges

To fully understand the regulatory mechanisms of Drp1, future research needs to employ various technical approaches, including gene editing, protein interaction analysis, and metabolomics, to reveal the functions of Drp1 under different physiological and pathological conditions. The widespread application of CRISPR/Cas9 gene editing technology allows researchers to quickly construct cell models to explore the roles of specific genes in the cell cycle and apoptosis. Additionally, the emergence of single-cell RNA sequencing technology enables researchers to analyze the dynamic changes in gene expression during the cell cycle and apoptosis at the single-cell level. Furthermore, exploring the combined application of Drp1 with other neurotoxic markers (such as Pink1 and Mfn2) will help improve the predictive and detection capabilities of propofol neurotoxicity and provide references for clinical translational research (Froehlich et al., 2024). The ways in which Drp1 influences Bax and Bcl-2 to initiate the caspase cascade reaction, thereby triggering neuronal apoptosis, also need to be explored in depth in the future. A critical question warranting further investigation is whether the combined use of anesthetic agents in clinical practice elicits differential Drp1-mediated effects on neuronal cells compared to propofol monotherapy.

## 8 Conclusion

Drp1 reduces the neurotoxicity of propofol by decreasing the production of ROS, adjusting the phosphorylation state of Drp1 to affect the cell cycle, regulating signaling pathways, and activating autophagy. However, the mechanisms and intrinsic relationships among these factors still require further investigation. There is considerable evidence from animal experiments and *in vitro* studies, but a lack of data at the human level. Although propofol exposure causes damage to developing hippocampal neurons, the mechanisms are not yet clear, but current research evidence points to changes in the expression of apoptosis-related proteins such as Bcl-2 and Bax, thereby affecting neuronal survival. However, this paper has limited clinical relevance and human data references, as it mainly relies on animal studies. As the research advances, we will better understand propofol’s neurotoxicity during human development and how Drp1 regulates this neurotoxicity.
